# Fetal death *in utero* and miscarriage in a patient with Crohn’s disease under therapy with ustekinumab: case-report and review of the literature

**DOI:** 10.1186/s12876-017-0633-6

**Published:** 2017-06-19

**Authors:** C. Venturin, S. Nancey, P. Danion, M. Uzzan, M. Chauvenet, C. Bergoin, X. Roblin, B. Flourié, G. Boschetti

**Affiliations:** 1 Department of Gastroenterology, Lyon-Sud hospital, Hospices Civils de Lyon, Université Lyon1, Lyon, France; 2 INSERM U1111, Centre International de Recherche en Infectiologie, Lyon, France; 3 Department of Gastroenterology and Nutritional Support, Beaujon Hospital, Assistance Publique des Hôpitaux de Paris, Université Paris VII, Clichy, France; 4 Department of Gastroenterology, Saint-Etienne hospital, Saint-Etienne, France; 5 Service d’Hépato-Gastroentérologie, Centre Hospitalier Lyon-Sud, 165 Chemin du Grand Revoyet, 69495 Pierre Benite, France

**Keywords:** Anti-IL12/23, Ustekinumab, Crohn’s disease, Pregnancy

## Abstract

**Background:**

Ustekinumab is a fully human monoclonal antibody against the p40 subunit of interleukin (IL) 12 and 23 which is involved in the pathogenesis of several inflammatory diseases. Ustekinumab is approved for psoriasis and psoriatic arthritis treatment and has been successfully evaluated in phase II and III trials for patients with Crohn’s disease (CD).

**Case presentation:**

We report here the case of a patient who became pregnant during treatment with ustekinumab for a refractory CD and which ended in miscarriage.

**Conclusion:**

Ustekinumab is a relatively new pharmacotherapy and in addition to this clinical case, we reviewed the published literature concerning the use of this treatment during pregnancy and its consequences on pregnancy and fetus outcome.

## Background

Ustekinumab, a fully human IgG1-κ monoclonal antibody directed against the p40 subunit shared by IL-12 and IL-23, inhibits the action of these 2 cytokines, which are critically involved in the pathogenesis of Crohn’s disease (CD). This novel biologic agent is currently approved in psoriasis and represents also a promising agent for the treatment of CD according to the efficacy and safety profile from recent pivotal phase II and III trials [1]. In addition, the efficacy of ustekinumab in CD has been reported in 3 different real-life retrospective studies [2–4]. Ustekinumab has been recently approved in France for a temporary recommendation of use in moderate to severe active CD with previous biologic therapy anti-TNF and anti-α4β7 integrin (vedolizumab) failures. As most of the female CD patients susceptible to require ustekinumab are of childbearing age and because ustekinumab is a relatively novel agent, its consequences on pregnancy and fetus outcome are a critical concern. Currently, available data regarding this issue – which mainly come from patients treated for psoriasis – remain scarce in the context of CD. We report here the case of a 32 year-old patient who became unexpectedly pregnant during treatment with ustekinumab for a refractory CD and ultimately had a miscarriage.

## Case presentation

We here report on a 32-year-old Caucasian female followed in our institution for a severe ileocolonic refractory CD since 2007 who had failed to respond to various therapies, including thiopurines, anti-TNF (infliximab and adalimumab) and to vedolizumab. Her obstetrical history included four pregnancies, one of which has ended by a miscarriage before 20 weeks and the 3 others were normal with 3 healthy children (gravidity 4 and parity 3, G4P3). Given severe flares of CD featured by abdominal cramping, chronic diarrhea, body weight loss despite oral steroids, ustekinumab was started in January 2016 (induction with subcutaneous (s.c.) injection of ustekinumab 90 mg at week 0, 2, 4) followed by a maintenance regimen with s.c. injections every 8 weeks. This led to a rapid clinical improvement and she quickly achieved a complete clinical and biological remission. In June 2016, after her 5th injection, she reported an unintended pregnancy. The last dose of ustekinumab had been administered at week 4 of gestation. Ultrasound (US) examination confirmed this inadvertent pregnancy and ustekinumab therapy was subsequently discontinued due to the lack of clinical data supporting this medication’s use during pregnancy. A systematic folic acid supplementation (1 mg/day) was started immediately according to French recommendations. In July 2016, while the patient was still in remission, a second US exam was performed and reported in utero fetal death (dating from week 4, close to the last ustekinumab injection) and ultimately leading to a miscarriage at 8 weeks of gestation.

## Discussion and conclusions

Up to now, the outcomes of inadvertent pregnancies occurring during ustekinumab therapy have been registered in only 65 cases of patients (most of them treated for psoriasis and only 3 for CD) [5–12]. All published cases are summarized in Table 1. Regarding the 3 cases of maternal exposures to ustekinumab during pregnancy that occurred in CD, 2 patients had also paradoxical psoriaform skin reactions and were treated with doses of ustekinumab, according to the indication for psoriasis (e.g. 45 mg s.c. every 12 weeks). In the present case, ustekinumab doses were higher both during induction and maintenance regimens (270 mg the first month followed by 90 mg every 8 weeks), as usually used in active refractory CD. Among all the published cases or unpublished data from clinical studies, spontaneous abortion was reported in 6 cases (9%), 16 cases of live births with no adverse event or congenital abnormality or birth defect, one case of live birth with premature baby with atrioventricular septal defect and right aortic arch and 2 cases of live birth associated with neonatal jaundice were reported. Among the 30 other cases out of the 65, in whom maternal exposure to ustekinumab during pregnancy was recorded, the pregnancy outcomes were unknown and finally in 10 cases, an elective termination of the pregnancy was performed. Ages of the pregnant women and additional risk factors of miscarriage as well as the duration of ustekinumab therapy during pregnancy were variable among the cases. In the present case, the patient was at increased risk of miscarriage since she had already experienced an obstetrical history of miscarriage in the past in the absence of ustekinumab exposure and given her age. As for psoriasis, CD raises by itself the risk of miscarriage especially during flare-ups of the disease [13].

**Table 1:** Pregnancy outcomes in ustekinumab-treated patients

Indication	Age (years old)	Outcome of pregnancy	Reference in the article
Pustular psoriasis and psoriatic arthritis	22	Uneventful	5
Psoriasis	35	Miscarriage (at 12 weeks of gestation)	6
Psoriasis	34	Uneventful	7
Psoriasis	24	Uneventful	7
Psoriasis	21	Uneventful	8
Psoriasis	25	Uneventful	9
Psoriasis	22	Uneventful	10
Psoriasis	29	Uneventful	10
Psoriasis	33	Unknown	10
Paradoxical psoriasis and Crohn’s disease	28	Uneventful	11
Crohn’s disease	37	Uneventful	12
Crohn’s disease	32	Miscarriage (at 8 weeks of gestation)	This report

Ustekinumab is an IgG_1_ monoclonal antibody that cannot cross the placenta by simple diffusion, but in contrast its active transport is mediated by the fetal Fc receptors expressed in the placenta. The expression of such Fc receptors is detected after 14 weeks of gestation and therefore the active transport of the biologic agent begins from the second trimester and increases rapidly till the end of pregnancy [14]. Interestingly, animal studies in pregnant monkeys, using high dose of ustekinumab, failed to demonstrate an enhanced risk for the foetus. Additionally no adverse event on pregnant females or foetuses have been noticed and the abortion rates were similar both in animals exposed to the drug compared to those without exposure [15, 16]. Ustekinumab has been subsequently classified as Pregnancy FDA category B, which means that there is no risk reported from animal studies; however, there are, up to date, no adequate and dedicated studies in women receiving ustekinumab during pregnancy. Interleukins 12 and 23 are implicated in the pathophysiology of CD but also have been involved in uterine physiology since they have been related to the impaired implantation of an embryo and subsequently development of the trophoblast [17]. These pro-inflammatory cytokines are specifically blocked by ustekinumab which may, at least theoretically, impact pregnancy by this way.

Collectively, all these data do not allow drawing final conclusions on the safety or the potential teratogenicity of ustekinumab in pregnancy due to the too small number of reported cases as well as their heterogeneity and given that spontaneous abortions often occur without knowledge of pregnancy. Gastroenterologists are faced with questions about the safety of this novel biologic agent during gestation. Teratogenic and abortion risks must be balanced with the risk of uncontrolled gut inflammation and complications in refractory CD. Even if animal studies using high doses of ustekinumab did not report teratogenicity and even if the risk of fetal drug-exposure may be outweighed by the clinical benefit for the mother, the uncertainty regarding potential risks should led the physician to avoid the use of ustekinumab in pregnant CD patients. Large registries and prospective dedicated studies are warranted in the future to definitively conclude.

## Abbreviations

CD: Crohn’s disease
S.c.: Subcutaneous
US: Ultrasound
